# Lifestyle management and brain MRI metrics in female Australian adults living with multiple sclerosis: a feasibility and acceptability study

**DOI:** 10.1186/s40814-024-01495-3

**Published:** 2024-05-02

**Authors:** Olivia Wills, Brooklyn Wright, Lisa-Marie Greenwood, Nadia Solowij, Mark Schira, Jerome J. Maller, Alok Gupta, John Magnussen, Yasmine Probst

**Affiliations:** 1 School of Medical, Indigenous and Health Sciences, University of Wollongong, Wollongong, NSW 2522 Australia; 2 School of Psychology, University of Wollongong, Wollongong, NSW 2522 Australia; 3 Illawara Health and Medical Research Institute, University of Wollongong, Wollongong, NSW 2522 Australia; 4 School of Medicine and Psychology, Australian National University, Canberra, ACT Australia; 5 General Electric Healthcare, Richmond, Melbourne, Australia; 6 Monash Alfred Psychiatry Research Centre, Melbourne, VIC Australia; 7 Wollongong Diagnostic Imaging Group, Wollongong, NSW Australia; 8 Faculty of Medicine, Health and Human Sciences, Macquarie University, Macquarie Park, NSW 2109 Australia

**Keywords:** Multiple sclerosis (MS), Lifestyle, Diet, Magnetic resonance imaging (MRI), Disability

## Abstract

**Background:**

Limited studies of multiple sclerosis (MS) exist whereby magnetic resonance imaging (MRI) of the brain with consistent imaging protocols occurs at the same time points as collection of healthy lifestyle measures. The aim of this study was to test the feasibility, acceptability and preliminary efficacy of acquiring MRI data as an objective, diagnostic and prognostic marker of MS, at the same time point as brain-healthy lifestyle measures including diet.

**Methods:**

Participants living with relapsing remitting MS partook in one structural MRI scanning session of the brain, completed two online 24-hour dietary recalls and demographic and self-reported lifestyle questionnaires (e.g. self-reported disability, comorbidities, physical activity, smoking status, body mass index (BMI), stress). Measures of central tenancy and level of dispersion were calculated for feasibility and acceptability of the research protocols. Lesion count was determined by one radiologist and volumetric analyses by a data analysis pipeline based on FreeSurfer software suite. Correlations between white matter lesion count, whole brain volume analyses and lifestyle measures were assessed using Spearman’s rank-order correlation coefficient.

**Results:**

Thirteen female participants were included in the study: eligibility rate 90.6% (29/32), recruitment rate 46.9% (15/32) and compliance rate 87% (13/15). The mean time to complete all required tasks, including MRI acquisition was 115.86 minutes $($± 23.04), over 4 days. Conversion to usual dietary intake was limited by the small sample. There was one strong, negative correlation between BMI and brain volume (*r*_s_ = −0.643, *p* = 0.018) and one strong, positive correlation between physical activity and brain volume (*r*_s_ = 0.670, *p* = 0.012) that were both statistically significant.

**Conclusions:**

Acquiring MRI brain scans at the same time point as lifestyle profiles in adults with MS is both feasible and accepted among adult females living with MS. Quantification of volumetric MRI data support further investigations using semi-automated pipelines among people living with MS, with pre-processing steps identified to increase automated feasibility. This protocol may be used to determine relationships between elements of a brain-healthy lifestyle, including dietary intake, and measures of disease burden and brain health, as assessed by T1-weighted and T2-weighted lesion count and whole brain volume, in an adequately powered sample.

**Trial registration:**

The study protocol was retrospectively registered in the Australia New Zealand Clinical Trials Registry (ACTRN12624000296538).

## Key messages regarding feasibility

- **What uncertainties existed regarding the feasibility?**
  Compared to bi-annual MRI as part of routine clinical care for people living with MS, the feasibility and acceptability of consistent MRI protocols as part of research studies are unknown. Our team investigated the recruitment, adherence to protocols, primary outcome measures, adverse events, feasibility of MRI data for semi-automated pipeline analysis and acceptability of acquiring MRI brain scans and measures of lifestyle at the same point in time, prior to upscaling the protocol.
- **What are the key feasibility findings?**
  The recruitment rate was 46.9% (15/32), eligibility rate 90.6% (29/32) and compliance rate 87% (13/15). The mean time to complete all tasks, including MRI acquisition, was 115.86 minutes $($± 23.04), over a period of 4 days. The MRI protocols were run through a semi-automated pipeline which produced significant processing errors in nine (69%) participants. This required manual intervention to correct the pial surface boundary and luminance inhomogeneity prior to achieving extraction of whole brain volume. There were no adverse events or safety concerns raised.
- **What are the implications of the feasibility findings for the design of the main study?**
  The results indicate that a large-scale study is feasible; however, the protocol evaluation highlights some modifications before progressing. An adequate sample size (with lifestyle measures such as dietary assessment as the primary outcome measure) should be considered in addition to higher quality and validated measures of lifestyle factors such as stress and sleep. Quality data and quantification of volumetric MRI data support further investigations using semi-automated pipelines to explore brain health in MS. The implementation of additional pre-processing steps is recommended to minimise post-processing errors. We also recognise that drawing definite conclusions from a single point in time is limited and does not provide enough insight to determine the upscale of this project, particularly when dealing with the complexities of a condition like MS. A longitudinal approach will provide greater insight into the protocol’s sustainability, effectiveness and impact on participant’s health behaviours. Powered studies will then clarify whether associations exist between brain-healthy lifestyle measures and/or dietary intake and measures of disability and brain health, including T1-weighted and T2-weighted lesion count and volumetric analysis.

## Introduction

### Background

Multiple sclerosis (MS) is a chronic, multi-faceted, neurological condition requiring unique considerations. Despite the therapeutic revolution of disease modifying therapies (DMTs) over the past decade [1], MS remains the most common disease of the central nervous system in young adults between 18 and 40 years [2]. Approximately 2.9 million people are diagnosed with MS globally, of whom over 33,300 live in Australia [2].

Despite knowledge of the established risk factors for MS, including genetic, environmental, immunological and infectious factors, the variability in clinical course between people living with MS (plwMS) remains poorly explained [3]. This variability necessitates a greater understanding of modifiable lifestyle factors that may reduce MS symptoms and improve quality of life outcomes, including levels of disability [4].

The impact of environmental factors such as physical activity, ultra-violet (UV) exposure and not smoking are vital in the daily management of MS and recent (2016) guidelines encourage plwMS to adopt a brain-healthy lifestyle to slow down the disease course and maximise outcomes [5]. Lifestyle management has also been shown to improve overall quality of life [6], empower plwMS to take an active role in the daily management of their disease, make informed choices and gain new perspectives for the collaborative development of person-centred care plans [7]. This is reflected in the Flinders Chronic Condition Management Program, a model of self-management where clients actively participate in decision-making to ensure interventions align with their values, priorities and beliefs [8]. This approach has seen many plwMS turn their attention towards lifestyle management.

Nutrition and diet is one emerging lifestyle element that is now being considered as one of the many possible factors that contribute to the pathogenesis of MS, with studies identifying distinct associations between dietary components and level of disability [9]. Previous reviews have found a high intake of saturated fat to be associated with poorer MS outcomes [9]. Supplementation of vitamin D and fatty acids have also shown to improve outcomes related to brain lesions and lower MS relapse rate [10]. Despite this single-nutrient focussed evidence, studies addressing whole ‘diets’ or patterns of eating that may improve or delay MS disability are lacking.

To date, large-scale clinical trial data exploring high-quality measures of disability and lifestyle have been limited to subjective measures of disability, inappropriate dietary methodology and rarely collect lifestyle measures at the same data collection time point [11, 12]. Such methods, therefore, rely on post hoc participant recall which has made it difficult to determine associations between lifestyle measures, including diet, and measures of MS disability [13]. Further, observational studies tend to use subjective measures to quantify disability for plwMS, such as the current gold standard and most popular assessment tool, the Kurtzke Expanded Disability Status Scale (EDSS) [14].

The EDSS is a neurologist-administered assessment scale evaluating functional systems of the central nervous system in MS [14]. PlwMS are assigned a score that corresponds to their level of ambulatory ability (ranging from 0: normal neurological exam/no disability to 10: death due to MS) in 0.5 increments [15]. Despite the tool being clinically validated and widely used in clinical studies, it has limited inter- and intra-rater reliability [16, 17] and does not capture any of the cognitive and/or ‘invisible’ elements of MS disability (i.e. chronic pain or fatigue). Many studies have also found the EDSS to lack sensitivity to changes in disease progression [18] which warrants need for neuroimaging to index markers of disease disability and therapeutic response among plwMS [19].

Magnetic resonance imaging (MRI) is a fundamental component of routine diagnostic, prognostic and medical management for plwMS, using magnetic fields and radio waves to measure the number and size of brain lesion(s) [20]. Integrating MRI information into MS monitoring has been shown to help predict the risk of long-term disability worsening [21], neurodegenerative phenomena and the evolution towards a more progressive phenotype in multiple large-scale, longitudinal and international studies [22]. Its role became central in the investigations of MS diagnosis and monitoring after the 1980s and it is now recommended, as a gold standard, that plwMS repeat a MRI every 6–12 months, or if new clinical symptoms occur, to monitor disease and its progression. This is because the presence of new and/or changing brain lesions and their association with disease relapse(s) generally develops over a period of 6 months.

To ensure a neurologist can accurately assess disease activity and progression in plwMS, the MRI scanner, image acquisition protocols and patient orientation must remain the same. However, such conditions rarely exist in clinical practice [23]. Further to this, limited studies in MS exist whereby these scans are conducted at the same time point as the collection of healthy lifestyle measures. Another challenge is variation of the processing and segmentation techniques when using a software suite for processing the brain images to obtain quality volumetric analysis to assess disability [24].

To the authors’ knowledge, no Australian study exists whereby objective measures of MS disability, using consistent MRI acquisition protocols and suitable lifestyle/dietary methodology, that considers the cognitive limitations of plwMS, has been conducted. Further, studies that have included diet/nutrition as one aspect of lifestyle management are limited.

### Aims and objectives

The primary aim of this study was to test the feasibility, acceptability and preliminary efficacy of acquiring MRI brain scans at the same time point as lifestyle measures, including dietary assessments, in a cohort of plwMS. The purpose of this study is to inform protocol designs of larger scale studies investigating the relationship between disease progression and the adoption of a brain-healthy lifestyle.

The specific objectives were to:

- Assess the *feasibility* of the study protocols by evaluating the proposed recruitment capability of the eligibility criteria and recruitment methods, participant retention rates and the quality and manual interventions of the semi-automated neuroimaging output for volumetric analysis.
- Assess the *acceptability* of data collection procedures by assessing participant burden through time to complete all study measures and protocol burden by the time required for quality control checks and manual editing of brain images.
- Ascertain the *preliminary efficacy* of collecting MRI and lifestyle/dietary intake measures in a cohort of plwMS, by determining the strength of association between dietary intake, exercise, body mass index (BMI), UV exposure, age, disease duration and (1) T2-weighted (T2w) white matter lesion count and (2) T1-weighted (T1w) volumetric analysis, using lifestyle profiles and MRI brain scans.

## Materials and methods

### Study design

A preliminary, cross-sectional analysis was conducted between March and September 2021 to investigate the feasibility, acceptability and preliminary efficacy of acquiring brain MRI and measures of lifestyle management among plwMS, at the same point in time. A local consumer group of patient and public contributors were involved from initial planning stages of the study design to dissemination of preliminary results and upscale conversations. Feasibility was defined as an overarching concept assessing the extent to which the research protocol can be successfully carried out, and acceptability was the extent to which people receiving/following the study procedures considered it to be appropriate based on the perceived amount of effort required to participate [25]. These methods were necessary to understand how the protocols were received, prior to repeated use or conducting on a larger study.

The ethics aspects of this study were approved by the University of Wollongong and the Illawarra Shoalhaven Local Health District’s Human Research Ethics Committee (Ref: 2021/ 001) and the research was conducted in accordance with the Helsinki Declaration to promote and protect the health of participants. The study is reported according to the Strengthening the Reporting of Observational Studies in Epidemiology (STROBE) checklist [26] and the Consolidated Standards of Reporting Trials (CONSORT) extension to pilot and feasibility trials [27]. The study protocol was retrospectively registered in the Australia New Zealand Clinical Trials Registry (ACTRN12624000296538). All participants provided written informed consent prior to commencing research participation.

### Participants

People living in New South Wales, Australia, diagnosed with a relapsing remitting MS (RRMS) phenotype were recruited between March and June 2021 via multimodal means including the MS Australia website, social media platforms including Facebook, Twitter and LinkedIn; snowball sampling and personal contacts. Telephone screening assessing eligibility included: (a) a self-reported diagnosis of a RRMS phenotype by a neurologist based on the McDonald Criteria [28], (b) aged $\ge$ 18 years, (c) able to read and communicate in the English language, (d) access to the Internet for dietary assessment measures and (e) understanding and willingness to comply with the study protocols. No formal sample size calculation was performed as the study was not designed to show statistical significance or power. The study aimed to recruit a minimum of 14 adults with MS to complete an in-depth analysis of MRI brain scans, as informed by previous reviews on adequate sampling in neuroimaging studies [29].

If participants met the inclusion criteria and provided informed consent, self-reported demographic (sex, date of birth) and clinical information including disease duration (years), time since last relapse (years) and MS medications was attained. Participants also self-reported their level of disability based on motor and neurological dysfunction using the Patient Determined Disease Steps (PDDS) which has been validated for use as a measure of physical disability in MS [30]. The PDDS scale is a simple, subjective measure of disability on a scale from 0 (normal; functionally normal with no limitations on lifestyle) to 8 (bedridden) [31]. It is a reproducible assessment of disability that can be undertaken by non-neurology specialists in MS and hence, the PDDS was utilised in this study as a surrogate of the EDSS [31].

### Data collection

Participants attended one single visit at the *Wollongong Diagnostics Centre* located in Corrimal, Australia, between April and May 2021 for their structural MRI (sMRI) scan and were asked to complete two, online, Automated Self Administered 24-hour (ASA24) dietary recalls, within the same week as their sMRI. During this visit, participants were also assessed with a structured questionnaire administered in interview format to obtain information on lifestyle management. Questions and lifestyle measures were adapted from the validated 2018 Lifestyle and Environment Survey developed by the Menzies Institute for Medical Research for the Australian MS Longitudinal Study (AMSLS) [32]. The AMSLS is the largest national cohort of plwMS, collecting self-reported surveys on diverse topics every year. This survey is widely used in MS research, ensuring reliability and comparability of data across studies. The protocols used for this research are detailed below.

#### MRI acquisition

All structural and diffusion MRI were obtained on a 3T GE Signa Architect MRI scanner (Waukesha, Milwaukee, USA) with a 48-channel head coil, at the *Wollongong Diagnostics Centre*, Corrimal, Australia. Neuroimaging sequences included a combination of two-dimensional (2D) and three-dimensional (3D) acquisition techniques to improve the realignment of anatomical orientation that is required to detect new brain lesions in plwMS [33, 34]. The MRI data were acquired as part of a 33-minute 05-second scan protocol.

T1w and several T2w MRI images were used to enable grey and white matter volumetric analyses based on FreeSurfer software [35] and to identify the presence of lesion(s) and their characteristics in brain tissue, respectively. The remainder of the image types, as described below, supported the collection of quality MRI data. The 2015 Magnetic Resonance Imaging in MS (MAGNIMS), 2016 Consortium of MS Centres (CMSC) and MRI Clinical Scientist (JM) guided the MRI protocols used in this study [24, 33].

sMRI types were used to provide quantitative information on size, shape, volume and integrity of brain structures and lesion characteristics. Diffusion weighted images (DWI) were used to measure the diffusion capacity of cellular tissue structures and are highly sensitive to acute and chronic brain tissue changes in plwMS. After acquiring a calibration and 2D three-plane localiser scan, the following nine structural MRI sequences were obtained:

- AC-PC aligned 3D sagittal *T1w IR-SPGR (BRAVO)* as 1 mm isotropic (repetition time (TR) = 0.008 ms, echo time (TE) = 0.003 ms, inversion time (TI) = 450 ms, flip angle (FA) = 12°, field of view (FOV) = 25.6 cm, number of excitations (NEX)=1, inplane resolution= 1mm x 1mm, matrix size= 256mm x 256mm, acquisition time= 4m 40s);
- 2D *T2w PROPELLER* in an (1) axial and (2) coronal orientation as 4-mm slice thickness (TR(1) = 8.475 ms, TR(2) = 4664 ms, TE(1) = 0.133 ms, TE(2) = 0.133 ms, FA = 142°, FOV = 22 cm, NEX = 1.5, inplane resolution (1,2) = 0.4297 mm × 0.4297 mm, matrix size (1,2) = 512 mm × 512 mm, acquisition time (1) = 2 m 23 s, (2) = 2 m 42 s);
- 3D sagittal *T2w FLAIR Cube* with 1-mm slice thickness (TR = 7000 ms, TE = 130 ms, TI = 1933 ms, HyperSense = 1.3, FA = 90°, FOV = 25.6 cm, NEX = 1, matrix size= 256mm x 256mm, inplane resolution = 1.0 mm × 1.0 mm, acquisition time = 4 m 37 s);
- 2D axial *T2w FLAIR* with 4-mm slice thickness (TR = 12,000 ms, TE = 140 ms, FA = 160°, FOV = 22 cm, NEX = 1, matrix size = 512 mm × 512 mm, inplane resolution = 0.5 mm × 0.5 mm, acquisition time = 2 m 36 s);
- 3D axial *Enhanced susceptibility weighted (eSWAN)* with 3-mm slice thickness (TR = 0.0555 ms, TE = 0.017 ms, FA = 15°, FOV = 22 cm, acquisition time = 2 m 57 s);
- 2D *DWI* in an axial orientation as 5-mm slice thickness (TR = 10,500 ms, TE = 0.0764 ms, FOV = 23.0 cm, matrix size = 256 mm × 256 mm, inplane resolution = 0.8984 mm × 0.8984 mm, total scan time = 1 m 3 s) and
- Axial *DWI HARDI acquisition*, with 140 diffusion directions (8 at *b* = 0, 25 directions at *b* = 700, 45 at *b* = 1000, 70 at *b* = 2800) 2.5-mm slice thickness (57 slices), matrix size = 100 mm × 100 mm, inplane resolution = 2.5 mm × 2.5 mm, TR = 4531 ms, TE = 0.103–0.115 ms, FOV = 25.0 cm, HyperBand = 3, ASSET = 2), with acquisition time of 11 m 15 s and for distortion correction an inverse blipped scan (6 at *b* = 0) with the same parameters (duration 41 s).

Image data were obtained in DICOM format and visually inspected for motion and severe anomalies by two researchers. To support reliability of the results, the MRI scanner, radiographers (RM, MN), patient orientation and image acquisition protocols (i.e. field strength and field of view, excitations, pulse sequence, spatial resolution) remained the same for all participants.

#### Lifestyle measures, including dietary assessment

An interview-based questionnaire was used to collect information on lifestyle measures including: smoking status (yes/no), duration of physical activity (minutes per day), BMI (weight/height(m)^2^), level of stress (0–4), UV exposure (minutes per day), vitamin D supplemental dose (IU), current dietary pattern and presence of comorbidities.

For high-quality dietary assessments, participants were emailed instructions that included a link and unique username and password to access the ASA 24-hour dietary recall Australian version 2016 tool, developed by the National Cancer Institute and adapted to the Australian food supply [36]. Participants were prompted via SMS to complete two diet recalls on non-consecutive days (one weekend day and one weekday) [37] using the ASA24 tool, within the same week as their sMRI scan. The ASA24 is a multi-pass recall methodology and guides participants through a series of questions to capture all food and beverage intake information within the previous 24-hour period. Data from validation studies indicates comparability between reported intakes obtained in interviewer-administered to self-administered dietary recalls [38] and has been used previously in MS populations [39].

ASA24 is guided by food inclusions and dietary supplement data files from the Australian Food, Supplement and Nutrient composition Database 2011–2013 that was developed in response to the 2011–12 National Nutrition and Physical Activity Survey [40]. Portion sizes were guided by those used in the 2011–13 Australian Health Survey [41]. The recall was not restricted to a completion time and respondents were able to complete their recall within multiple sittings if required.

### Outcomes

The Conceptual Framework for Implementation Outcomes by Proctor et al. was used to guide the language and describe the outcomes of implementation evaluated in this study. These outcomes are described in detail below [42].

#### Feasibility and acceptability

The recruitment capabilities were assessed by recruitment rate (number responded to recruitment methods/number provided written informed consent), time taken for informed consent to be attained, time taken to meet required sample size, effective recruitment strategies, reason for denial or ineligibility to participate and sufficient or limiting nature of the eligibility criteria (number eligible/number completed initial screen) [25, 43, 44]. To assess the quality of the reconstructed image output using semi-automated pipelines with this protocol, FreeSurfer (Secondary outcomes section) outlines a quality control process which involved visual checks for error(s) in the semi-automated segmentation process such as skull stripping, white matter or pial surface errors. When necessary, manual edits of the pial surface, white matter volume and topological defects are recommended [45].

Acceptability of data collection procedures among the intended individuals were assessed by compliance rate (number of participants in the final analysis/number enrolled at commencement of study), participant burden (time taken to complete the dietary recalls, total time of MRI scans, number of participants requiring rescan due to MRI errors, number of participants rescanned due to error, distance travelled to receive MRI, total number of tasks to complete, total time to complete required tasks and number of participants requiring assistance with required tasks) [25, 46, 47] and the time taken to complete quality assessment and editing of MRI images using FreeSurfer software.

#### Secondary outcomes

##### Manual assessment of lesion characteristics

One radiologist (AG) visually inspected the axial and sagittal T2w images to manually determine total lesion count, their dimensions and report the location of each lesion [48]. To do this, the radiologist scrolled through continuous sections in the axial and sagittal plane whilst visually inspecting the images. A lesion was defined as an area of focal hyperintensity, being > 5 mm in at least one dimension. Lesion *location* was reported as an important measure to track disease progress, including, for future studies, where new lesions appear over time or how the size of lesions in each location progress [49]. Total lesion count for each participant is reported under the preliminary efficacy outcomes. Gladionium enhancement was not used to detect active lesions.

##### Automated brain segmentation methods

MRI were converted from DICOM to NIfTI format using dcm2niix on MRIcron cross-platform format image viewer [50]. A step-wise directive script was used to instruct the cortical reconstruction and volumetric segmentation stream of the T1-W.nii.gz images to run, using a data analysis pipeline based on FreeSurfer software suite (http://surfer.nmr.mgh.harvard.edu/), version 7.1.1 [45]. FreeSurfer is an online imaging programme for the analysis and visualisation of structural neuroimaging data [51, 52] which accounts for motion correction of multiple T1w images and segmentation of white and grey matter volumetric structures [53]. The technical details of these procedures are described elsewhere [54].

The ‘recon-all’ processing command within FreeSurfer, with default parameters, was used to construct white and grey matter surface estimates from the T1-W.nii.gz 3D anatomical volume with multiple intermediate steps such as luminance inhomogeneity, bias field corrections and skull stripping. Thereafter, total grey matter volume (GMV) (left hemisphere cortical GMV, right hemisphere cortical GMV, subcortical GMV, total cortical GMV), total cerebral white matter volume (WMV) (left hemisphere WMV, right hemisphere WMV) and whole brain volumes were estimated [55].

To demonstrate the feasibility of analysing these MRI protocols using semi-automatic segmentation pipelines with high quality output, the reconstructed T1w.nii.gz images were visually inspected in Freeview, FreeSurfer’s visualisation tool. All T1w images were examined in a slice-by-slice view checking for errors in borders (i.e. white matter, grey matter and pial surface borders), subcortical segmentation, intensity normalisation, skull strip and topological defect [45]. The images that did not pass the initial quality check were manually edited/corrected (e.g. by the addition of manual control points, erasing voxels or using the recon-edit tool to remove dura from the brainmask file) and the segmentation process was repeated from the pial edit stage, as suggested by FreeSurfer guidelines [45].

##### Dietary analysis

The repeated dietary recall data were used to calculate usual dietary intake using the multiple source method (MSM) [56]. Usual nutrient intakes were calculated for individual participants using a three-step statistical procedure to account for between person and within person variation. Firstly, the probability of consuming the nutrient of interest (response variable) on a particular day was estimated for each participant. Secondly, the usual amount of the macronutrient or micronutrient was estimated on consumption days. Finally, to estimate the usual daily intake for each participant, responses from step one and two were multiplied.

Response variables included total energy, protein, total carbohydrates, total fat, dietary fibre and total sugars. Analysis of retinol, B12 iron, calcium and magnesium were also included in the analysis due to their potential associations with MS disability [10, 57, 58]. Response variables were adjusted for overall energy intake and estimated assuming that all participants were habitual consumers, with age and sex as explanatory variables that influence the MSM regression model. Usual intake data were compared to the estimated average requirement (EAR) and upper limit (UL) according to the Nutrient Reference Values 2006 for Australia and New Zealand [59]. Over- and/or underreporting of dietary intake was not assessed in these methods.

#### Data analysis

All statistical analysis was completed using IBM SPSS Statistics (V. 25.0, 2017, IBM Corp). Measures of spread including the median, interquartile range, mean and standard deviation were calculated for the primary research aim and objectives regarding feasibility and acceptability of research protocols. The Shapiro–Wilk test was applied to assess the normality of the data and reported accordingly. Descriptive statistics were completed for all participant demographic characteristics and lifestyle measures including dietary intake. Scatterplots between dietary, lifestyle, PDDS, age, disease duration and MRI outcomes (lesion count and whole brain volume) were visually inspected for monotonicity and where a monotonic relationship existed were variables assessed using the Spearman’s rank-order correlation coefficient (signified by *r*_s_). This is a nonparametric measure of the strength and direction of association between two variables. These results are narratively explored in this manuscript. A significant result was considered at *p* < 0.05.

## Results

### Participants

**Fig. 1:**
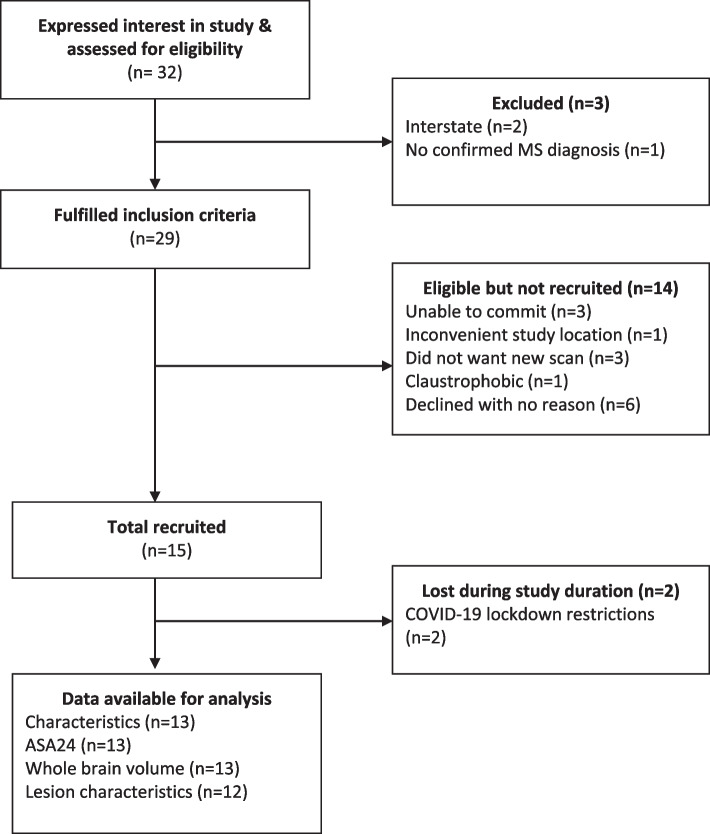
Study participant’s CONSORT diagram

Overall, 32 people expressed an interest in the study and were screened for eligibility, of which 29 met the inclusion criteria. Of these, 15 consented and completed the pre-screen questionnaire (Fig. 1). There were no formal withdrawals during the data collection period. However, two participants were excluded from final data analysis due to an inability to obtain MRI data as a result of the COVID-19 NSW, Australia lockdown requirements during July to September 2021. Therefore, 13 participants completed all measures and their questionnaire responses, lifestyle measures including two ASA24 responses, PDDS score and axial and sagittal T1w and T2w MRI data were analysed. Lesion characteristics were available for *n* = 12 participants due to the delay in data collection for one participant as a result of COVID-19.

### Characteristics of the participants

Demographic and lifestyle characteristics (Table 1) indicate that the sample was entirely female (*n* = 13). The mean age of participants was 44.62 years (± 7.65) and the mean BMI (underweight: < 18.5, healthy weight: 18.5–24.9, overweight: 25–29.9, obese: > 30) was 26.62 kg/m^2^ (± 5.61), indicating majority of the sample were overweight. Four participants (31%) were classified as obese.

**Table 1:** Baseline demographic and lifestyle measures of participants

**Characteristics**	**Participants with MS (*n* = 13)**
Female	13 (100%)
Age, years	44.62 (± 7.65)^a^
Disease duration, years	13.85 (± 10.69)^a^
PDDS	2 (0–3)
Time since last relapse, years	1.2 (0.92–8)
BMI, kg/m^2^	26.62 (± 5.61)^a^
Smoking status—number	
Never smoked	12 (92%)
Current smoker	0 (0%)
Ex-smoker	1 (8%)
Physical activity—daily minutes	60 (30–85)
Stress/anxiety^b^	2.57 (1.5–4)
Vitamin D supplement—IU	4000 (500–17,500)
UV exposure—daily minutes	30 (20–60)
MS medications—number	
None	2 (15%)
Ocrevus infusion	3 (23%)
Tysabri infusion	3 (23%)
Copaxone infusion	2 (15%)
MAVENCAD	1 (8%)
Aubagio	1 (8%)
Fingolimod	1 (8%)

Baseline descriptive statistics for plwMS. Data reported as median (interquartile range) for normally distributed data and frequency (percentage of total sample) for discrete data

*PDDS* Patient Determined Disease Steps, *BMI* body mass index, *UV* ultra-violet, *MS* multiple sclerosis

^a^Shapiro–Wilk test *p* > 0.05—mean (SD) is reported

^b^Stress and anxiety reported on a scale from: 0, *never* stressed or anxious to 4, *very often*

Mean disease duration was 13.85 years (± 10.69), median time since the last relapse was 1.2 years (IQR 0.92–8) and the median PDDS score at screening was 2: moderate disability (IQR 0–3). Eight (62%) participants were using infusion-based DMTs, three (23%) oral DMTs and the remaining two (15%) were not on any DMT. All participants were non-smokers at the time of screening. The median time spent physically active per day was 60 minutes (IQR 30–85) and the median time spent in the sun per day was 30 minutes (IQR 20–60).

At the time of the study, 85% of participants (*n* = 11) were taking at least one nutrition supplement. Vitamin D was the most used supplement at 91% (*n* = 10), followed by fish oil 27% (*n* = 3), vitamin B12 27% (*n* = 3) and iron 18% (*n* = 2). More than half (54%, *n* = 7) of participants followed a ‘special diet:’ two followed the overcoming MS diet (no dairy or meat and low fat), one followed a gluten-free Swank diet (low saturated fat), one followed a gluten-free paleo diet (no processed foods, sugar or trans fat), two excluded red meat (one of these participants also excluded fish from their diet) and one followed an anti-inflammatory diet (favouring fruit and vegetables, whole-grains, lean proteins and omega-3).

### Outcome data

#### Feasibility

##### Recruitment capability

This study had a recruitment rate of 46.9% (*n* = 15/32) and an eligibility rate of 90.6% (*n* = 29/32). The median time taken to obtain written informed consent following provision of study information was one day (IQR 1–11) and the proposed sample size was reached after 3 months of recruitment.

Three participants were not eligible to participate due to being located interstate (*n* = 2) and one was without a clinically definite diagnosis of MS. An additional 14 participant enquiries did not result in participation. The main reasons being unable to commit due to timing of data collection periods (*n* = 3), did not want an additional MRI on top of routine scans though could provide an existing scan (*n* = 3), inconvenient study location (*n* = 1), claustrophobia (*n* = 1) and six declined without reason.

**Fig. 2:**
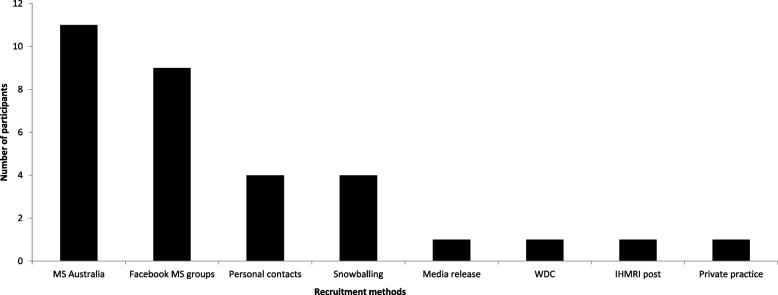
Effectiveness of recruitment strategies leading to interest in the study

**Fig. 3:**
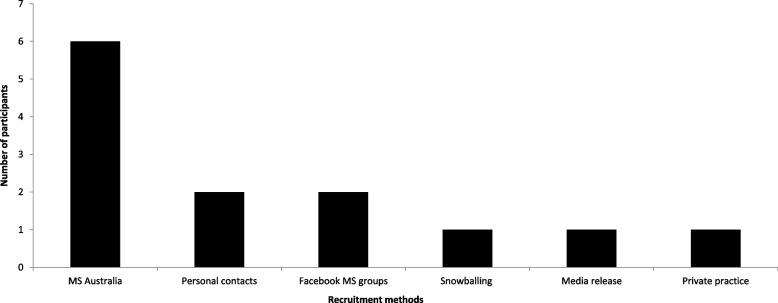
Effectiveness of recruitment strategies leading to informed consent

Of the 32 participants who expressed initial interest in the study, the MS Australia website was the most popular recruitment platform with 34.3% (*n* = 11) (Fig. 2). This was followed by 28.1% (*n* = 9) of people made aware of the study from posts in Facebook MS groups and 12.5% (*n* = 4) via personal contacts. The most popular recruitment method among those who provided informed consent was the MS Australia website (40%) (Fig. 3).

##### Retention

Complete data were obtained from all 13 participants with a confirmed MRI appointment. Two additional participants were scheduled to attend their MRI scan and complete the ASA24 tool during COVID-19 lockdown, however were unable to attend the scanner due to lockdown restrictions limiting their ability to leave their local government area. This converted to a compliance rate of 87% (*n* = 13/15).

##### Quality of neuroimaging data

**Fig. 4:**
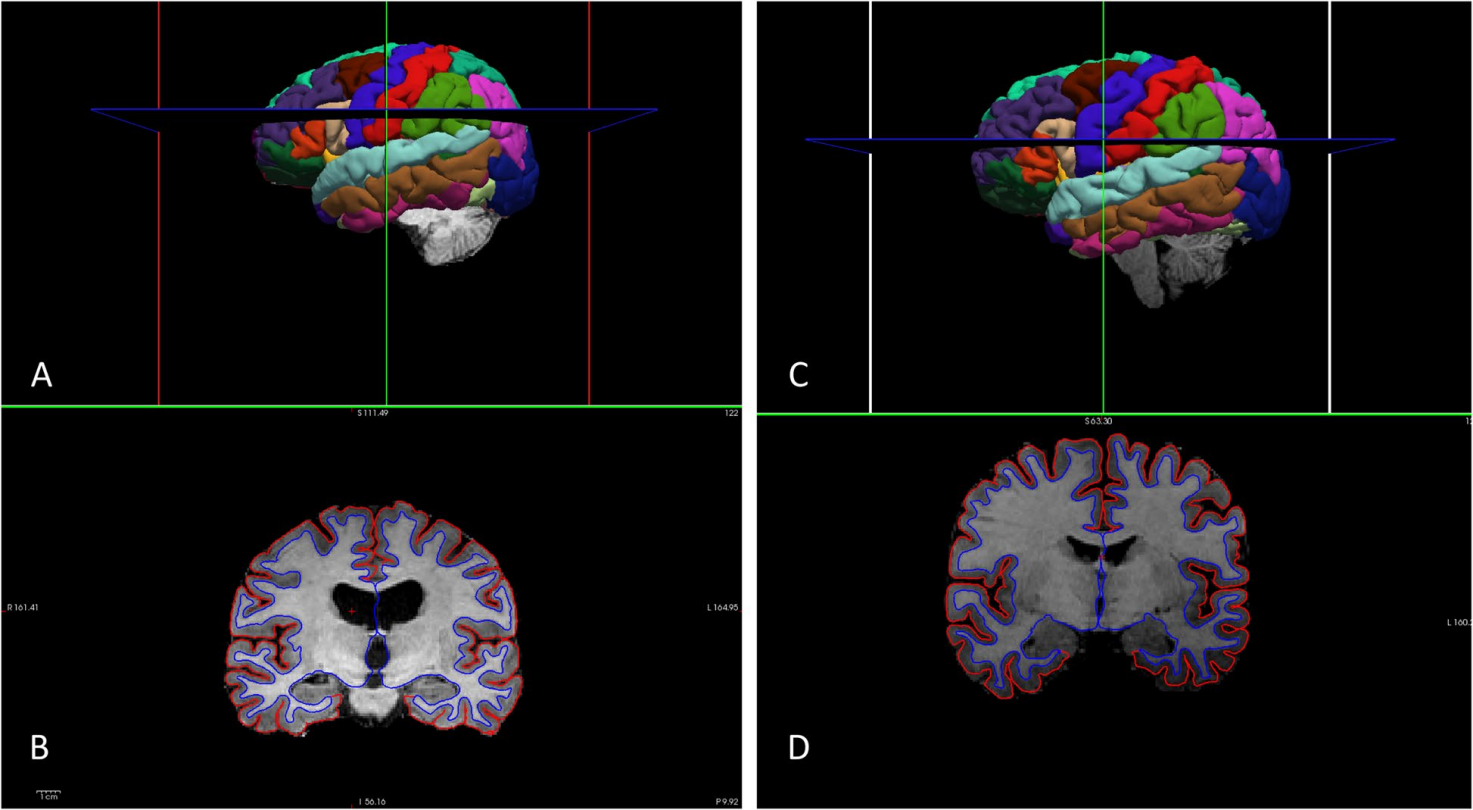
Quality T1w brain-reconstruction using FreeSurfer software suite, indicating correct pial surface boundaries and surface segmentation. **A**, **C** The manually edited T1w brain image in sagittal plane shows an accurate identification of brain region segmentation and **B**, **D** reconstructed T1w brain image in the coronal plane shows an accurate boundary and subcortical segmentation

Of the 13 participant T1w reconstructed brain MRI images, 2 (15%) passed initial quality control checks with no manual analysis steps required (Fig. 3). Luminance inhomogeneity was identified for five participants (38%). Following N4 bias field correction, an algorithm used for correcting non-uniform intensity present in MRI image data, this data passed quality control checks. Four participants (31%) had persistent errors in the pial surface boundary that were not corrected following N4 bias field correction and were, therefore, manually corrected using a brain mask created using hb-bet [60] and rogue voxels were removed via the mrcalc command from the MRtrix3 software package [61]. Despite errors, these additional steps increased automated feasibility and produced usable data for all *n* = 13 participants (Fig. 4).

#### Acceptability

##### Time to complete study measures

The median time taken to complete the first dietary recall using the ASA24 tool was 43 minutes (IQR 33–57) and the median time to complete the second recall was 38.5 minutes (IQR 29–46). In total, 54% (*n* = 7) of participants took less than 40 minutes to provide their dietary recall.

The median number of food items recalled in the first ASA24 was 22 (IQR 20–30) and the median number in the second recall was 20 (IQR 16–27). Of the 13 participants who completed the ASA24, 92% (*n* = 12) completed without assistance and 8% (*n* = 1) completed the recall in an interview style due to technical difficulties logging in to their online account.

The average time to complete all six required tasks in the study (demographic questionnaire, PDDS, safety questionnaire, lifestyle measures, MRI, two dietary recalls) was 115.86 minutes (± 23.04), over a period of 4 days. No serious or adverse events were reported beyond typical clinical descriptions within this population.

Only two participant images passed initial quality control checks and did not require further manual intervention. Of the remaining 11, the time required for manual editing ranged from 60 minutes in images with only minor errors (*n* = 6) to over 2.5 hours in images with significant errors (*n* = 5), taking approximately 70 minutes, on average.

#### Preliminary efficacy

The mean total number of lesions among *n* = 12 participants from all brain regions on a T2w, 3D FLAIR image was 21.17 (± 9.19). Lesions were primarily found in the occipital and temporal lobes, followed by the parietal lobe. The average whole brain volume (BrainSegVol: including cerebellum and ventricles but not including the dura) for *n* = 13 participants was 1,074,928.62 mm^3^ (± 83,961.36), average white matter volume was 3726.36 mm^3^ (± 2253.42), average subcortical grey matter volume (SubCortGrayVol: including the thalamus, caudate, putamen, pallidum, hippocampus, amygdala, accumbens, ventral DC and substantia nigra) was 50,526.69 mm^3^ (± 4470.15) and the average total grey matter volume (TotalGrayVol) was 583,470.97 mm^3^ (± 43,463.01). The average human brain volume is ~1,130,000 mm^3^ in adult women, although there is substantial variation [62].

Table 2 summarises the participants’ usual macronutrient and micronutrient intake data (from food and beverages) as estimated from the ASA24 dietary recalls, compared to the acceptable macronutrient distribution range (AMDR). The percentage of total fat contributing to overall energy intake (38%) was slightly above the AMDR, whereas the percentage of carbohydrates contributing to overall energy intake (39%) was below the AMDR. Protein intake was adequate. Usual nutrient intake of all micronutrients exceeded the estimated average requirements for sex and gender. Magnesium was the only micronutrient that exceeded the upper limit of 350 mg/day.

**Table 2:** Usual dietary intake from ASA24 recalls

**Nutrient**	**Unit**	**ASA24 recall (*n* = 13)**	**% TE**	**AMDR**
**Energy**	kJ/day	8322.32 (± 2053.51)		
**Protein**	g/day	89.14 (± 27.56)	18%	10–35%
**Total fat**	g/day	84.90 (± 28.94)	38%	20–35%
**Carbohydrates**	g/day	189.20 (± 643.83)	39%	45–65%
**Sugar**	g/day	88.40 (IQR 80.6–100.9)		
**Dietary fibre**	g/day	30.60 (± 10.85)^a^		
**Iron**	mg/day	12.86 (± 4.61)^a^		
**Vitamin C**	mg/day	107.88 (IQR 69.83–185.83)^a^		
**Calcium**	mg/day	856.93 (± 349.26)^b^		
**Magnesium**	mg/day	412.18 (± 135.71)^c^		
**Retinol**	$\upmu$ g/ day	1124.20 (IQR 652.49–1755.51)^a^		
**Vitamin B12**	$\upmu$ g/ day	3.75 (± 2.27)^a^		

*ASA24 Automated Self-Administered 24-Hour Dietary Recall, TE* Total energy, *AMDR* Acceptable macronutrient distribution range

Shapiro–Wilk test indicated *p* > 0.05—mean (SD) is reported. *p* < 0.05—median (IQR) is reported

^a^Exceeded EAR for women aged 31–50 and 51–70

^b^Exceeded EAR for women 31–50 years only

^c^Exceeded UL

Monotonic correlations between lifestyle measures, including dietary intake, age, disease duration, T2w white matter lesion count and T1w whole brain volume are outlined in Table 3. There was one strong, negative correlation found between BMI and brain volume that was statistically significant (*r*_s_ = −0.643, *p* = 0.018) and one strong, positive correlation between physical activity and brain volume that was also statistically significant (*r*_s_ = 0.670, *p* = 0.012). A number of weak to moderate correlations were also found between: (1) lesion count and retinol, physical activity and UV exposure and (2) whole brain volume and dietary fibre, calcium, retinol and age.

**Table 3:** Spearman’s rank-order correlation coefficient between lifestyle measures and brain MRI metrics

**Lifestyle measure**	**Lesion count (*n* = 12)**	**Whole brain volume (*n* = 13)**
**Dietary intake**		
**Carbohydrate**		−0.027 (0.929)
**Fibre**		0.236 (0.437)
**Calcium**		−0.280 (0.354)
**Magnesium**	−0.067 (0.837)	
**Retinol**	−0.256 (0.422)	−0.429 (0.144)
**Physical activity**	0.284 (0.372)	0.670 (0.012)*
**BMI**		−0.643 (0.018)*
**UV exposure**	−0.241 (0.451)	
**Age**		−0.298 (0.323)
**Disease duration**		0.019 (0.95)

Only those variables that met the assumption of monotonicity were correlated using the Spearman’s rank-order coefficient, i.e. *r*_s_ value (sig *P* value)

*BMI* body mass index, *UV* ultra-violet

^*^Sig result at *p* < 0.05

## Discussion

The primary aim of this clinical study was to test the feasibility, acceptability and preliminary efficacy of acquiring MRI brain scans at the same time point as measures of lifestyle management, including diet, among plwMS. To the authors’ knowledge, this was the first Australian study whereby objective indices of MS disability, using consistent between-subject MRI protocols, high-quality dietary intake tools and suitable lifestyle methodology were conducted. The recruitment, retention, compliance rates and participant times to complete the study protocols indicated that the recruitment capability and data collection protocol were both feasible and accepted by participants living with MS.

The preliminary efficacy findings provide early evidence to support further investigations exploring the relationship between lifestyle management and outcomes of MS disability including lesion load and whole brain volume. Quantification of volumetric MRI data support further investigations using semi-automated pipelines among plwMS, with pre-processing steps identified in order to minimise post-processing errors. In a real-world and clinical setting, integrating MRI imaging with lifestyle assessments at the same point in time hold promise for enhancing effective management planning that encompasses behavioural change support, as well as personalised interventions that are unique to individual circumstances. Powered studies will clarify whether associations exist between lifestyle measures including dietary intake and measures of disability.

All participants were adult females, interested in nutrition and followed general healthy lifestyle self-management behaviours such as avoiding cigarettes and participating in regular physical activity in line with the exercise training guidelines for MS published in 2019 [63]. As the study had a strong focus on lifestyle management in MS, it may have encouraged individuals who were motivated and those that actively engaged in healthy lifestyle management practices, leading to potential volunteer bias.

Despite a wide range in the disease duration reported by participants (1 to 37 years), the sample showed an overall moderate level of physical disability (PDDS 2). The participants’ clinical characteristics can be partially explained in the context of the eligibility criteria, which restricted participants with primary or secondary progressive MS phenotypes from participating, thereby limiting participants with advanced disease and disability [64]. Further, almost all participants were taking DMTs (*n* = 11) which are associated with a reduced risk of increasing levels of disability, as previously reported in a randomised control trial [65]. Therefore, results of this study cannot be generalised beyond the criteria of this study.

The number of people who expressed initial interest in the study (*n* = 32) within 3 months was reasonable in the context of the proposed sample size, and the eligibility rate of 90.6% was high. This may be attributed to the in/exclusion criteria which only restricted three participants who were located interstate (*n* = 2) and one that had no confirmed MS diagnosis. Overall, the recruitment rate of 46.9% (4.3 participants per month) was significantly lower than the average recruitment rate reported in other published Australian studies involving plwMS [66]. One study exploring exercise self-management interventions reported a recruitment rate of 13 participants per month [66]. This suggests that the recruitment period of this study may need to be extended for future, larger studies involving lifestyle/diet and MRI measures for plwMS with over recruitment to cater for participant eligibility following expressed interest.

The reasons that eligible participants decided not to provide informed consent can provide an insight into aspects of the study design that may need to be improved prior to upscaling. Two factors that were consistently found to influence participation was the requirement for an additional ‘research MRI’ and the confusion that MRI posed a radiation risk. The majority questioned whether an existing scan could be provided as an alternative, or whether they were able to add on their neurologists’ referral to the scan time, to reduce the number of MRIs needed within a 1-year period. Participants were also worried that an additional MRI would increase radiation exposure, despite safety information provided to participants explaining that MRIs have no risk of exposure to radiation. Providing participants with additional information about the difference between research and clinical MRI scans may facilitate further recruitment numbers to overcome this barrier [67].

Previous poor scan experience and claustrophobia was predicted to be a barrier to recruitment; however, only one participant reported claustrophobia and refused to participate. This may be attributed to the information provided to participants upon expressing interest in the study. The information outlined measures implemented to minimise stress and anxiety including providing headphones with music and a security buzzer to all participants. Additionally, due to the nature of the study, participants who found MRIs to be distressing and uncomfortable likely did not express initial interest in the study. Moreover, the location of the study was another barrier to participation in a minority of participants (*n* = 3) and could potentially be overcome through expanding data collection to multiple sites, if identical scanning parameters could be ensured, prior to upscaling.

Overall, the time taken (3 months) to reach the proposed sample size of 14 was longer than anticipated. To address this, it was expected that the recruitment phase would need to be prolonged to reach the proposed sample size of a larger-scale study, within a given period. Of the recruitment methods, a majority of participants were made aware of the study via the MS Australia website (*n* = 13) and Facebook groups (*n* = 9). With MS Australia being the main resource portal for both plwMS and researchers, these findings were expected.

Recruitment through MS outpatient clinics and the establishment of collaborative neurology teams is consistently reported as an effective strategy to increase recruitment rates among plwMS [68]. However, this was not considered as a strategy in the present study due to the lack of a local MS clinic. Focus groups conducted within neurology treatment areas have identified a common theme suggesting the important role of the treating physician in influencing a person’s decision to participate in clinical research. It is also commonly reported that recruitment via this method can increase the eligibility rate, as the neurologist will only identify eligible participants [68]. Furthermore, four out of the eight proposed recruitment methods required the Internet and/or a social media presence, potentially limiting older adults or adults from underserved communities from participating. Therefore, to shorten the recruitment duration, increase recruitment and eligibility rates, and maintain homogeneity of the sample will require collaboration with neurology teams potentially at multiple sites.

Despite 85% of the reconstructed T1w brain images requiring post-processing intervention, the manual editing procedures and interventions used in this study do reflect the errors identified by the FreeSurfer instructions for quality control [45, 69]. However, the average edit time was significantly longer compared to FreeSurfer’s estimations of 30 minutes [69]. This time difference may be partially explained by 69% of the data having pial surface boundary errors which are more time-consuming to correct and/or the experience of the researcher with FreeSurfer error identification and corrections. The large number of manual corrections required warrant considerations given to pre-processing stages prior to the recon-all command to increase the automated feasibility.

There were no formal participant withdrawals throughout the study; however, due to the COVID-19 global pandemic, two participants were unable to undertake MRI scans and one participant’s scan was significantly delayed due to lockdown restrictions. Therefore, MRI and lifestyle profiles were obtained for 13 participants (87% of the consenting cohort). The rate of ‘withdrawal’ (13%) is lower compared to other Australian observational studies that use MRI data [70, 71]. These studies have reported withdrawal rates of 20–40% among plwMS. Therefore, this can indicate a high degree of acceptability of the study protocols and was somewhat expected given the small number of tasks required by participants.

The median time (40.75 minutes) taken for the participants to complete one 24-hour dietary recall using the ASA24 online tool was longer than what has been previously reported. Data show that US respondents completed their 24-hour dietary recall using ASA24 within an average of 24 minutes, and most completed their recall within 17–34 minutes [72]. Further to this, a second study by Kupis et al. [66] revealed the average time taken for participants to complete the ASA24 was 27.4 minutes. The results of the present study were somewhat expected, given that cognitive impairment occurs in 40–65% of plwMS and is characterised by hindered attention skills, information processing speed and concentration [73]. In addition, the average number of food items recalled (*n* = 21) was higher than what has been previously reported which may explain the increased time to complete the dietary recalls [72]. It is also recognised that there is a learning effect apparent with the use of online tools and the dietary recall was used for the first time for a majority of the participants (*n* = 12). Computer literacy and/or cognitive burden of using the online dietary assessment tool also needs to be considered as a potential issue prior to upscaling the study design.

Reported in a 2021 preliminary study exploring the validity of self-reported dietary assessments in adults with MS were solutions to improve the ASA24 protocols [74]. Participants with MS have suggested keeping a food diary in parallel with the ASA24 recalls to reduce the burden of recall memory, or queried an option for continuous ASA24 entries throughout the day, to reduce the burden of recall time. These suggestions would change the form of dietary methodology from one of usual to actual intake which has implications to its interpretation. In line with the conclusions of Silverira et al. [74], further research is required before considering these practices in a larger study to ensure that knowledge of the recall day does not influence dietary intake.

Overall, the average time to complete all six required tasks in this study indicated a low participant burden and wide acceptance of study protocols. FreeSurfer instructions state that manual corrections should take no more than 30 minutes; however, this timeframe was exceeded to ensure careful quality assessment and editing [68]. We suspect that the number of errors per image and researcher experience with FreeSurfer affected this outcome.

Lesion characteristics from our study were consistent with what has been reported in other studies. Curti et al. [75] reported a mean value of T2w FLAIR lesions as 22.7 (± 16.42) per participant, which is near identical to the findings in this current study being 21.17 (± 9.19). The locations where lesions were identified have also been referred to as common among MS cohorts [76]. Upscaling and broadening of the eligibility criteria to participants with a more progressive form of MS may produce different results.

Whilst brain volumes are usually measured and reported over time in MS to support brain atrophy rates and calculations of brain volume loss, this manuscript supports the use of FreeSurfer to calculate whole brain volume and grey matter and white matter volume changes over time for future studies (Fig. 4). The average whole brain volume 1,074,928.62mm^3^ (± 83,961.36) among participants was lower compared to the average human brain for adult females (~1,130,000 mm^3^) [62]. These results were expected given that brain volume loss is known to occur throughout the MS disease and at a rate faster than the general population [77]. In addition, given that the MRI protocols took 33 minutes and other pre-scan questionnaires were collected which required the participants to be fasted, we cannot rule out that hydration status may have had an impact on the MRI metrics.

Due to the unpowered sample size, it was expected that no significant correlations would be found between lifestyle measures and clinical variables. However, there was one strong, negative correlation found between BMI and brain volume that was statistically significant (*r*_s_ = −0.643, *p* = 0.018), suggesting an association between increasing BMI with decreasing brain volume. These results are largely reflected in the literature with increasing evidence stating that overweight and obesity is associated with chronic inflammation, worsening disability and poorer MS outcomes [78]. Further, the second positive, statistically significant association between physical activity and brain volume (*r*_s_ = 0.670, *p* = 0.012), has also been widely established [79]. Therefore, education on obesity management that forefronts the importance of healthy eating and physical activity, as an integral components of MS management, is gaining increased traction and should be highlighted as non-pharmacologic interventions to improve clinical outcomes of MS. The number of weak to moderate correlations warrant further investigation into the effects of specific elements encapsulating lifestyle management on disease outcomes and progression. We must here acknowledge that the risk of type 2 error in interpreting the correlation analyses is high due to the small sample. Scatterplots were assessed for monotonicity though challenged finding precise monotonic relationships between variables. Appropriate sample size calculations should be conducted prior to upscaling to determine the number of participants required to detect a significant association and to improve the rigor of these tests.

The MSM, as opposed to other methods for estimating usual nutrient intake, such as the National Cancer Institute method, has shown to be more reliable and enabled more precise estimates for the 10^th^ and 90^th^ percentiles when working with small sample sizes (*n* < 150) [80]. Therefore, a significant strength of the study protocols was the use of the validated MSM for calculating usual nutrient intake from repeated 24-hour dietary recalls, though the sample of our study was notably small for in-depth dietary analysis.

### Limitations

Despite the limited sample size (*n* = 13), the number of participants in neuroimaging studies is generally smaller, compared to studies exploring dietary assessment/lifestyle measures. A 2020 review exploring appropriate sample sizes in over 880-neuroimaging studies reported the median sample size to be 6 and the mean 8 [81]. Furthermore, 82% of the included studies had a sample size ≤ 10. A second review evaluating the sample size from ~1500 of the most cited MRI papers found that 96% of MRI studies had a median sample size of 12 [29]. This is due to the large number of MR images obtained, as was reflected in this study. Again, adequate sample size calculations should be conducted prior to upscaling, with lifestyle measures such as diet, rather than imaging, as the primary variable. As the sample size increases, this should also help to improve the reliability of the results.

Despite assessing the acceptability of study protocols through data collection measures, participants were not directly asked for feedback on the tools and methods followed. Future studies should include an acceptability assessment and consider additional valid and reliable measures of assessing implementation [42] to provide a more comprehensive understanding of the protocol’s real-world applicability. Finally, the ASA24 does not include data for omega-3 and vitamin-D and specific neurotransmitters such as choline, which have been previously reported to be associated with MS disability [10]. Alternative tools may need to be considered if associations between these nutrients are to be assessed in future studies. Further to this, the use of regression models to consider confounding factors that influence disability in MS should be considered when exploring potential relationships between MRI measures, including the addition of lesion volume and lifestyle profiles in a larger sample. Consideration of participation in rehabilitation programmes could be added to the eligibility criteria if these procedures are upscaled to a powered intervention study.

We acquired more data than presented here, specifically the advanced diffusion MRI. This is a focus of future investigations whereby white matter metrics and tractographic analyses will be performed and presented, as will the voxel-based data from FreeSurfer, in the context of MS and dietary intake.

## Conclusion

The initial results of this study suggest that acquiring a comprehensive suite of MRI brain scans at the same time point as measures of lifestyle management is feasible and acceptable to adult participants living with MS. Quality data and quantification of volumetric MRI data support further investigations using semi-automated pipelines among plwMS, with pre-processing steps to minimise post-processing errors and increase automated feasibility. Prior to upscaling, collaboration with consumers such as neurologists should be considered to shorten the recruitment duration, increase the recruitment rate and eligibility rate, whilst maintaining homogeneity of the sample.

Preliminary associations have highlighted the importance of education on obesity management that forefronts the importance of healthy eating and physical activity to improve clinical outcomes of MS. Ultimately, this type of study has the potential to identify relationships between elements of a brain-healthy lifestyle, including dietary intake, and measures of disease burden and brain health, as assessed by T1w and T2w lesion count and whole brain volume, in an adequately powered sample. The use of regression models that include confounders that influence disease progression should be considered.

## Data Availability

The datasets used and/or analysed during the current study are available from the corresponding author on reasonable request.

## Abbreviations

MS: Multiple sclerosis
DMT: Disease modifying therapy
plwMS: People living with MS
RRMS: Relapsing remitting MS
MRI: Magnetic resonance imaging
EDSS: Expanded disability status scale
PDDS: Patient determined disease steps
ASA24: Automatic self-administered 24-h dietary recall
T1w: T1-weighted
T2w: T2-weighted
2D: Two-dimensional
3D: Three-dimensional
sMRI: Structural MRI
DWI: Diffusion weighted image
MSM: Multiple source method
BMI: Body mass index
WMV: White matter volume
GMV: Grey matter volume
AMDR: Acceptable macronutrient distribution range
EAR: Estimated average requirement
UL: Upper limit

## References

[1] Hauser SL, Chan JR, Oksenberg JR (2013). Multiple sclerosis: prospects and promise. Ann Neurol.

[2] MS Australia. Multiple sclerosis rising and accelerating in Australia new data shows. 2023. Available from: https://www.msaustralia.org.au/news/multiple-sclerosis-rising-and-accelerating-in-australia-new-data-shows/.

[3] National Multiple Sclerosis Society. What causes MS? National Multiple Sclerosis Society; 2020. Available from: https://www.nationalmssociety.org/What-is-MS/What-Causes-MS.

[4] National Multiple Sclerosis Society. Diet, exercise & health behaviours. National Multiple Sclerosis Society; 2020. Available from: https://www.nationalmssociety.org/Living-Well-With-MS/Diet-Exercise-Healthy-Behaviors.

[5] Giovannoni G, Butzkueven H, Dhib-Jalbut S (2016). Brain health: time matters in multiple sclerosis. Mult Scler Relat Disord.

[6] Wills O, Probst YC (2022). Understanding lifestyle self-management regimens that improve the life quality of people living with multiple sclerosis: a systematic review and meta-analysis. Health Qual Life Outcomes.

[7] Lorig K (1993). Chronic disease self-management: a model for tertiary prevention. Generations.

[8] Flinders University. Flinders program information paper. 2017. Available from: https://www.flindersprogram.com.au/wp-content/uploads/Flinders-Program-Information-Paper.pdf.

[9] Sand IK (2018). The role of diet in multiple sclerosis: mechanistic connections and current evidence. Neurol Dis Cogn Funct.

[10] Tredinnick AR, Probst YC (2020). Evaluating the effects of dietary interventions on disease progression and symptoms of adults with multiple sclerosis: an umbrella review. Adv Nut.

[11] Simpson S, Tan H, Otahl P, Taylor B, Ponsonby AL, Lucas RM (2016). Anxiety, depression and fatigue at 5- year review following CNS demyelination. Acta Neurol Scan.

[12] Gascoyne CR, Simpson S, Chen J, van der Mei I, Marck CH (2019). Modifiable factors associated with depression and anxiety in multiple sclerosis. Acta Neurol Scand.

[13] Marck CH, Probst Y, Chen J (2021). Dietary patterns and associations with health outcomes in Australian people with multiple sclerosis. Eur J Clin Nutr.

[14] Gray O, Butzkueven H (2008). Measurement of disability in multiple sclerosis. Neur Asia.

[15] MS Trust. Expanded disability status scale (EDSS). MS Trust; 2020. Available from: https://mstrust.org.uk/a-z/expanded-disability-status-scale-edss.

[16] Meyer-Moock S, Feng YS, Maeurer M, Dippel FW, Kohlmann T (2014). Systematic literature review and validity evaluation of the Expanded Disability Status Scale (EDSS) and the Multiple Sclerosis Functional Composite (MSFC) in patients with multiple sclerosis. BMC Neurol.

[17] Roche. Disease progression in multiple sclerosis. Roche; 2016. Available from: https://www.roche.com/dam/jcr:a34e1a9e-47eb-4656-8ff4-69ae48979e8b/en/MS-disease-progression.pdf.

[18] Vaney C, Blaurock H, Gattlen B, Meisels C (1996). Assessing mobility in multiple sclerosis using the rivermead mobility index and gait speed. Clin Rehabil.

[19] McFarland HF (2009). Examination of the role of magnetic resonance imaging in multiple sclerosis: a problem-orientated approach. Ann Indian Acad Neurol.

[20] National Multiple Sclerosis Society. Magnetic resonance imaging (MRI). National Multiple Sclerosis Society; 2020. Available from: https://www.nationalmssociety.org/Symptoms-Diagnosis/Diagnosing-Tools/MRI#section-0.

[21] Filippi M (2020). Identifying progression in multiple sclerosis: new perspectives. Ann Neurol.

[22] Rocca MA, Valsasina P, Meani A, Pagani E, Cordani C, Cervellin C, Filippi M (2021). Network damage predicts clinical worsening in multiple sclerosis: a 6.4-year study. Neurol Neuroimmunol Neuroimmflamm.

[23] Erbayat E (2013). Reliability of classifying multiple sclerosis disease activity using magnetic resonance imaging in a multiple sclerosis clinic. JAMA Neurol.

[24] Sastre-Garriga J, Pareto D, Battaglini M, Rocca MA, Ciccarelli O, Enzinger C, MAGNIMS study group (2020). MAGNIMS consensus recommendations on the use of brain and spinal cord atrophy measures in clinical practice. Nat Rev Neurol.

[25] Orsmond GI, Cohn ES (2015). The distinctive features of a feasibility study: objectives and guiding questions. OTJR.

[26] Vandenbroucke JP, von Elm E, Altman DG, Gotzsche PC, Multor CD, Pocock SJ (2007). Strengthening the Reporting of Observational Studies in Epidemiology (STROBE): explanation and elaboration. PLoS Med.

[27] Lancaster GA, Thabane L (2019). Guidelines for reporting non-randomised pilot and feasibility studies. Pilot Feasibility Stud.

[28] MS Research Australia. How is MS diagnosed? MS Research Australia; 2020. Available from: https://msra.org.au/news/how-is-ms-diagnosed/.

[29] Szucs D, Loannidis JPA (2020). Sample size evolution in neuroimaging research: an evaluation of highly-cited studies (1990–2012) and of latest practices (2017–2018) in high-impact journals. NeuroImage.

[30] Learmonth YC, Motl RW, Sandroff BM (2013). Validation of patient determined disease steps (PDDS) scale scores in persons with multiple sclerosis. BMC Neurol.

[31] Marrie RA, Goldman M (2007). Validity of performance scales for disability assessment in multiple sclerosis. Mult Scl J.

[32] Alfredsson L, Olsson T (2019). Lifestyle and environmental factors in multiple sclerosis. Cold Spring Harb Perspec Med.

[33] Wattjes (2021). 2021 MAGNIMS-CMSC-NAIMS consensus recommendations on the use of MRI in patients with multiple sclerosis. Lancet Neurol.

[34] Hu XY, Rajendran L, Lapointe E (2019). Three-dimensional MRI sequences in MS diagnosis and research. Mult Scler.

[35] Lloyd-Jones G. MRI interpretation: T1 V T2 images. Radiology Masterclass; 2017. Available from: https://www.radiologymasterclass.co.uk/tutorials/mri/t1_and_t2_images.

[36] NIH. Automated self-administered 24- hour (ASA24) dietary assessment tool. National Cancer Institute; 2021. Available from: https://epi.grants.cancer.gov/asa24/.

[37] Dietary Assessment Primer. 24-hour dietary recall (24HR) at a glance. NIH National Cancer Institute. Available from: https://dietassessmentprimer.cancer.gov/profiles/recall/. Accessed 9 June 2021.

[38] Thompson FE, Dixit-Joshi S, Potischman N, Dodd KW, Kirkpatrick SI, Kushi LH (2015). Comparison of interviewer-administered and automated self-administered 24-hour dietary recalls (ASA24) in three diverse integrated health systems. Am J Epidemiol.

[39] Guan V, Simpson-Yap S, Nag N, Jelinek G, Neate S, Probst Y (2022). Using online 24-h dietary methodology to validate the psychometric properties of a dietary scoring tool with an international sample of adults living with multiple sclerosis. Nutrients.

[40] FSANZ. AUSNUT 2011-2013. Food Standards Australia New Zealand; 2020. Available at: https://www.foodstandards.gov.au/science/monitoringnutrients/ausnut/pages/default.aspx.

[41] ABS. Micro data and TableBuilder: Australian Health Survey: Nutrition and Physical Activity. Data from the National Nutrition and Physical Activity Survey 2011-2012 component of the Australian Health Survey 2011-13. 2018. Available at: https://www.abs.gov.au/statistics/microdata-tablebuilder/available-microdata-tablebuilder/australian-health-survey-nutrition-and-physical-activity.

[42] Proctor E (2011). Outcomes for implementation research conceptual distinctions, measurement challenges, and research agenda. Adm Policy Ment Health.

[43] Doody P, Lord JM, Whittaker AC (2019). Assessing the feasibility and impact of an adapted resistance training intervention, aimed at improving the multi-dimensional health and functional capacity of frail older adults in residential care settings: protocol for a feasibility study. Pilot Feasibility Stud.

[44] O’Dwyer JL, Russell AM, Bryant LD (2018). Developing and feasibility testing of data collection methods for an economic evaluation of a supported self-management programme for adults with a learning disability and type 2 diabetes. Pilot Feasibility Stud.

[45] FreeSurfer. Introduction to FreeSurfer output. Harvard; 2022. Available from: https://surfer.nmr.mgh.harvard.edu/fswiki/FsTutorial/OutputData_freeview.

[46] Noble NE, Paul CL, Carey ML, Sanson-Fisher RW, Blunden SV, Stewart JM (2014). A cross-sectional survey assessing the acceptability and feasibility of self-report electronic data collection about health risks from patients attending an Aboriginal Community Controlled Health Service. BMC.

[47] Tapsell LC, Thorne R, Batterham M, Russel J, Ciarrochi J, Peoples G (2016). Feasibility of a community based interdisciplinary lifestyle intervention trial on weight- loss (the HealthTrack study). Nut Diet.

[48] Filippi M, et al. Assessment of lesions on magnetic resonance imaging in multiple sclerosis: practical guidelines. J Neuro. 2019;142(7):1858–1875. 10.1093/brain/awz144 PMC6598631 31209474

[49] Bodini B, Battaglini M, De Stefano N (2011). T2 lesion location really matters: a 10 year follow-up study in primary progressive multiple sclerosis. J Neurol Neurosurg Psychiatry.

[50] NITRC. MRIcron V1.0.20190902. 2019. Available from: https://www.nitrc.org/projects/mricron.

[51] Reuter M, Schmansky NJ, Rosas HD, Fischl B (2012). Within-subject template estimation for unbiased longitudinal image analysis. Neuroimage.

[52] Segonne F (2004). A hybrid approach to the skull stripping problem in MRI. Neuroimage.

[53] Fischl B (2004). Sequence-independent segmentation of magnetic resonance images. Neuroimage.

[54] Dale AM, Fischl B, Sereno MI (1999). Cortical surface-based analysis. I. Segmentation and surface reconstruction. Neuroimage.

[55] Ontandeda D, Fox RJ (2017). Imaging as an outcome measure in multiple sclerosis. Neurotherapeutics.

[56] Harttig U, Haubrock J, Knüppel S (2011). The MSM program: web-based statistics package for estimating usual dietary intake using the Multiple Source Method. Eur J Clin Nutr.

[57] Goldberg P, Fleming MC, Picard EH (1986). Multiple sclerosis: decreased relapse rate through dietary supplementation with calcium, magnesium and vitamin D. Med Hypotheses.

[58] Hagemeier J, Tong O, Dwyer MG, Schweser F, Ramanathan M, Zivadinov R (2015). Effects of diet on brain iron levels among healthy individuals: an MRI pilot study. Neurobiol Ageing.

[59] National Health and Medical Research Council, Australian Government Department of Health and Ageing, New Zealand Ministry of Health (2006). Nutrient reference values for Australia and New Zealand.

[60] Isensee F, Schell M, Tursunova I, Brugnara G, Bonekamp D, Neuberger U, et al. Automated brain extraction of multi-sequence MRI using artificial neural networks. Hum Brain Mapp. 2019;40(17):4952–4964. 10.1002/hbm.24750 PMC6865732 31403237

[61] Tournier JD, Smith RE, Raffelt D, Tabbara R, Dhollander T, Pietsch M (2019). MRtrix3: a fast, flexible and open software framework for medical image processing and visualisation. Neuroimage.

[62] Zaidi Z (2010). Gender differences in human brain: a review. Anatomy.

[63] Kim Y (2019). Exercise training guidelines for multiple sclerosis, stroke, and Parkinson disease: rapid review and synthesis. Am J Phys Med Rehabil.

[64] MS Australia. Types of MS. Australia; 2017. Available from: https://www.msaustralia.org.au/about-ms/types-ms.

[65] Claflin SB, Broadley S, Taylor BV (2019). The effect of disease modifying therapies on disability progression in multiple sclerosis: a systematic overview of meta-analyses. Front Neurol.

[66] Kupis J, Johnson S, Hallihan G, Olstad D (2019). Assessing the usability of the automatic self- administered dietary assessment tool (ASA24) among low-income adults. Nutrients.

[67] Committee for Protection of Human Subjects. Magnetic resonance imaging (MRI) in research. Berkeley: University of California; 2021. Available from: https://cphs.berkeley.edu/mri.pdf.

[68] Lindblad AS, Zingeser P, Sismanyazici-Navaie N (2011). Incentives, and barriers to neurological clinical research participation. Clin Investig.

[69] FreeSurfer. Troubleshoot your output. Harvard; 2022. Available from: https://surfer.nmr.mgh.harvard.edu/fswiki/FsTutorial/TroubleshootingData.

[70] Kjølhede T, Siemonsen S, Wenzel D, Stellmann JP, Ringgaard S, Pedersen BG (2018). Can resistance training impact MRI outcomes in relapsing-remitting multiple sclerosis?. Mult Scler.

[71] Langeskov-Christensen M, GrøndahlHvid L, Nygaard MKE, Ringgaard S, Jensen HB, Nielsen HH (2021). Efficacy of high-intensity aerobic exercise on brain MRI measures in multiple sclerosis. Neurology.

[72] Subar AF, Potischman N, Dodd KW, Thompson FE, Baer DJ, Schoeller DA (2020). Performance and feasibility of recalls completed using the Automated Self-Administered 24-Hour Dietary Assessment Tool in relation to other self-report tools and biomarkers in the interactive diet and activity tracking in AARP (IDATA) Study. J Acad Nutr Diet.

[73] Jongen PJ, Ter Horst AT, Brands AM (2012). Cognitive impairment in multiple sclerosis. Minerva Med.

[74] Silveira SL, Jeng B, Gower BA, Motl RW (2021). Feasibility, acceptability, and preliminary validity of self-report dietary assessment in adults with multiple sclerosis: comparison with doubly labeled water measured total energy expenditure. Nutrients.

[75] Curti E, Graziuso S, Tsantes E, Crisi G, Granella F (2018). Correlation between cortical lesions and cognition impairments in multiple sclerosis. Brain Behav.

[76] Kister I. The multiple sclerosis lesion checklist. Prac Neurol. 2018;17(6):68–73.

[77] Radue EW (2015). Correlation between brain volume loss and clinical and MRI outcomes in multiple sclerosis. Neurology.

[78] Lutfullin I, Eveslage M, Bittner S, on behalf of the German Competence Network Multiple Sclerosis (KKNMS) (2023). Association of obesity with disease outcome in multiple sclerosis. Neurol Neurosurg Psychiatry.

[79] Klaren RE (2015). Objectively measured physical activity is associated with brain volumetric measurements in multiple sclerosis. Behav Neurol.

[80] Laureano GHC, Torman VBL, Crispim SP, Dekkers ALM, Camey SA (2016). Comparison of the ISU, NCI, MSM and SPA methods for estimating usual intake: a simulation study of nutrients consumed daily. Nutrients.

[81] Hanspach J, Nagel AM, Hensel B, Uder M, Koros L, Laun FB (2020). Sample size estimation: current practice and considerations for original investigations in MRI technical development studies. MR Med.

